# Plasma levels of matrix metalloproteinase-2, -3, -10, and tissue inhibitor of metalloproteinase-1 are associated with vascular complications in patients with type 1 diabetes: the EURODIAB Prospective Complications Study

**DOI:** 10.1186/s12933-015-0195-2

**Published:** 2015-03-10

**Authors:** Stijn A Peeters, Lian Engelen, Jacqueline Buijs, Nish Chaturvedi, John H Fuller, Casper G Schalkwijk, Coen D Stehouwer, EURODIAB Prospective Complications Study Group

**Affiliations:** Department of Internal Medicine, Maastricht University Medical Centre, P.O. Box 5800, 6202 AZ Maastricht, the Netherlands; Department of Internal Medicine, Atrium Medical Centre, Heerlen, the Netherlands; Institute of Cardiovascular Sciences, University College London, London, UK; Department of Epidemiology and Public Health, University College London, London, UK

**Keywords:** Type 1 diabetes, Matrix metalloproteinase, Tissue inhibitor of metalloproteinase, Cardiovascular disease, Albuminuria, Retinopathy

## Abstract

**Background:**

Impaired regulation of extracellular matrix remodeling by matrix metalloproteinases (MMPs) and tissue inhibitor of metalloproteinase (TIMP) may contribute to vascular complications in patients with type 1 diabetes. We investigated associations between plasma MMP-1, −2, −3, −9, −10 and TIMP-1, and cardiovascular disease (CVD) or microvascular complications in type 1 diabetic patients. We also evaluated to which extent these associations could be explained by low-grade inflammation (LGI) or endothelial dysfunction (ED).

**Methods:**

493 type 1 diabetes patients (39.5 ± 9.9 years old, 51% men) from the EURODIAB Prospective Complications Study were included. Linear regression analysis was applied to investigate differences in plasma levels of MMP-1, −2, −3, −9, −10, and TIMP-1 between patients with and without CVD, albuminuria or retinopathy. All analyses were adjusted for age, sex, duration of diabetes, Hba1c and additionally for other cardiovascular risk factors including LGI and ED.

**Results:**

Patients with CVD (n = 118) showed significantly higher levels of TIMP-1 [β = 0.32 SD (95%CI: 0.12; 0.52)], but not of MMPs, than patients without CVD (n = 375). Higher plasma levels of MMP-2, MMP-3, MMP-10 and TIMP-1 were associated with higher levels of albuminuria (p-trends were 0.028, 0.004, 0.005 and 0.001, respectively). Severity of retinopathy was significantly associated with higher levels of MMP-2 (p-trend = 0.017). These associations remained significant after further adjustment for markers of LGI and ED.

**Conclusions:**

These data support the hypothesis that impaired regulation of matrix remodeling by actions of MMP-2, -3 and-10 and TIMP-1 contributes to the pathogenesis of vascular complications in type 1 diabetes.

**Electronic supplementary material:**

The online version of this article (doi:10.1186/s12933-015-0195-2) contains supplementary material, which is available to authorized users.

## Background

Type 1 diabetes is associated with an increased risk of macro- and microvascular disease [1,2]. The exact mechanisms leading to vascular damage in type 1 diabetes have not been fully established [3], but impaired regulation of extracellular matrix (ECM) remodeling by matrix metalloproteinases (MMPs) may contribute to the development of vascular complications [4,5]. Increased MMP activity has been associated with increased matrix turnover and potentially to pathological reorganization of the ECM in atherosclerosis, aneurysm formation, plaque disruption [4] and, in diabetic nephropathy, to glomerular basement membrane thickening [5].

MMPs are a group of zinc- and calcium-dependent endopeptidases, which degrade and rebuild proteins of the ECM, such as collagen, elastin, gelatin and casein [6]. Thus far, more than 20 different MMPs have been identified, which, according to their various functions, can be divided into collagenases (e.g. MMP-1), gelatinases (MMP-2 and MMP-9), stromelysines (e.g. MMP-3 and MMP-10), matrilysines, and others. These enzymes can be excreted by various cells (e.g. fibroblasts, endothelial cells, monocytes and macrophages) or can be incorporated in the cellular membrane. Tissue inhibitors of metalloproteinases (TIMP-1-4) and α2-macroglobulin inhibit the action of MMPs [6].

Plasma levels of MMPs have been observed to be higher in individuals with type 1 diabetes, compared to controls [7-9]. The association between circulating MMPs and macrovascular disease in patients with type 1 diabetes has not been investigated, while studies on the associations between MMP levels and microvascular complications seem contradictory. For example, high levels of serum MMP-9 and a high MMP-9-to-TIMP ratio have been related to retinopathy [10], and high levels of plasma MMP-10 have been associated with nephropathy and proliferative retinopathy [8]. In contrast, other studies did not show significant associations between plasma levels of MMP-2, -9 or TIMP-1 and microvascular complications [7,11]. In fact, most studies were rather small [7,9-11] or limited to only one or two MMPs [7-11]. In one large study of 269 type 1 diabetes patients and 269 non-diabetic controls [8], plasma MMP-10 was associated with nephropathy and proliferative retinopathy. However, plasma TIMP was not measured, whereas this may be of importance as MMP-10 activity is dependent on the amount of inhibitors. Nevertheless, in type 1 diabetes, hyperglycaemia, low-grade inflammation (LGI) and endothelial dysfunction (ED) have been found to be associated with higher plasma and tissue levels of MMPs and TIMP [5,9,12], and LGI and ED are consistently associated with macro- and microvascular complications [13-15].

In view of these considerations, we hypothesized that high plasma levels of MMPs and TIMP are related to macro- and microvascular complications in individuals with type 1 diabetes, possibly through associations with LGI and ED. We investigated these hypotheses in a cross-sectional study of patients with type 1 diabetes, in whom we assessed macro- and microvascular disease and measured plasma levels of MMP-1, −2, −3, −9, −10 and TIMP-1, as well as biomarkers of LGI and ED.

## Methods

### Study population

In this analysis, data were used from the EURODIAB Prospective Complications Study, a European prospective cohort study that has been described in detail previously [16,17].

In brief, baseline inclusion was performed between 1989 and 1991 in 3,250 patients with type 1 diabetes. Inclusion criteria were defined as a clinical diagnosis of type 1 diabetes before the age of 36 years, and requirement of continuous insulin therapy within the first year of diagnosis. Patients aged between 15 and 60 years were recruited from 31 centres in 16 European countries. Sample selection was stratified by sex, age group and duration of diabetes to ensure sufficient representation of all categories. These patients were invited for a follow-up visit 7–9 years after baseline enrolment. Of the 3,250 included patients, 1,880 (57.8%) returned for re-examination. At follow-up, a cross-sectional nested case–control study was performed in a subset of patients (n = 543). Cases (n = 348) were those with one or more complications and controls (n = 195) were those with no evidence of complications [18]. This type 1 diabetic patient control group was used to investigate differences in plasma levels of MMPs and TIMP-1 compared with type 1 diabetic patients with vascular complications. For the current analyses, clinical data, plasma samples for analysis of MMPs and TIMP-1 and information about potential confounders and mediators were available in 493 of these 543 patients. The study was approved by the local Ethics Committee at each centre and all patients gave informed consent.

### Main determinants

Concentrations of MMPs and TIMP-1 were determined in plasma samples that were stored at −80°C after collection and in our laboratory until analyses and they were never thawed previously. Plasma levels of MMP-1, MMP-2, MMP-3, MMP-9, MMP-10 and TIMP-1 were measured using a commercially available enzyme-linked immunosorbent assay (ELISA) kit [Human MMP 3-Plex Kit (for MMP-1, −3 and −9), Human MMP-2-Plex Kit (for MMP-2 and -10) and Human TIMP-1 Kit, MSD, Rockville, United States of America] according to the manufacturer’s protocol. Plasma samples of 10, 25 and 2.5 microliter were used for the MMP-3-plex kit, MMP-2-plex kit and TIMP-1 kit, respectively. The MMPs were detected in both a pro- and an active form. TIMP-1 was detected only in the active form. The intra- and inter-assay coefficients of variation were 7.0% and 8.0% for MMP-1, 4.5% and 5.9% for MMP-2, 8.4% and 12.3% for MMP-3, 5.3% and 8.9% for MMP-9, 4.4% and 9.7% for MMP-10, and 4.3% and 5.2%for TIMP-1, respectively.

### Main outcomes

#### Macrovascular disease

Cardiovascular disease (CVD) was defined as a cardiovascular event in a patient’s medical history, including myocardial infarction, angina, coronary artery bypass graft, stroke or ischaemic changes in a centrally Minnesota-coded ECG [19].

#### Microvascular disease

Albumin excretion rates were measured from duplicate 24-hour urine collections [17]. Micro- and macroalbuminuria were defined as an albumin excretion rate between 20 and 200 μg/min, or above 200 μg/min, respectively. We also estimated the glomerular filtration rate (eGFR) using the Chronic Kidney Disease Epidemiology Collaboration (CKD-EPI) equation [20].

Retinopathy was assessed from retinal photographs according to the EURODIAB protocol; non-proliferative retinopathy was defined as the presence of one or more microaneurysms, hemorrhages, and/or hard exudates. Proliferative retinopathy was defined as presence of any new vessels, fibrous proliferations, pre-retinal haemorrhages, vitreous hemorrhages or photocoagulation scars [21].

### Other variables

Apart from demographic data, additional information about medication, smoking history, systolic blood pressure and duration of diabetes was collected. Body weight and height were measured in patients wearing indoor clothing without shoes. With these values body mass index (BMI) was calculated. Fasting blood samples were taken for measurements of lipid profile and glycemic control. Cholesterol and triglyceride levels were measured by enzymatic colorimetric tests [22], HDL was measured directly [23]. Friedewald’s formula was used to calculate LDL levels [24]. Glycated hemoglobin (HbA1c) was measured by a latex-enhanced turbidimetric immunoassay (Roche Products, Welwyn Garden City, UK). The reference range for this assay was 4.2-6.2%.

### Markers of LGI

Plasma levels of C-reactive protein (CRP) were measured with a highly sensitive ELISA, developed and validated in our own laboratory [18]. Plasma levels of interleukin-6 (IL-6) and tumor necrosis factor-α (TNF-α) were measured using commercially available ELISA kits (R&D Systems, Oxon, U.K.) [18]. Intra- and inter-assay coefficients of variation were 3.9 and 8.7% for CRP, 4.5 and 9.0% for IL-6, and 7.3 and 8.5% for TNF-α, respectively.

### Markers of ED

Plasma soluble E-selectin (sE-selectin) and soluble vascular cell adhesion molecule-1 (sVCAM-1) were measured by sandwich enzyme immunoassays (R&D Systems, OXON, U.K.) in duplicate. Individual mean values of the duplicates were used in the ED scores. Intra- and inter-assay coefficients of variation were 2.1% and 3.1% for sE-selectin, and 4.0% and 9.1% for sVCAM-1, respectively.

### Statistical analyses

All analyses were performed using the Statistical Package for Social Sciences (SPSS), version 20 (IBM Corporation, Armonk, NY, USA). Log transformation was performed for variables with a skewed distribution (triglycerides, serum creatinine, eGFR, MMP-1, MMP-2, MMP-3, MMP-9, MMP-10, CRP, IL-6 and TNF-α). Student’s t- or Chi-Square tests were performed for comparisons of characteristics between individuals with and without vascular complications, as appropriate.

Linear regression analyses were performed to examine the extent to which plasma levels of MMP-1, MMP-2, MMP-3, MMP-9, MMP-10 and TIMP-1 differed between individuals with and without CVD or microvascular complications as well as to test for linear trends in plasma levels of MMPs and TIMP-1 between both micro- and macroalbuminuria and between non-proliferative and proliferative retinopathy. Results of these analyses are presented as standardized regression coefficients to enable comparison of the magnitude of the associations. We have only investigated single MMPs and TIMP-1 in the analyses and have not adjusted for the presence of others, because each MMP has its own substrate specificity and actions.

Analyses were adjusted for age, sex, duration of diabetes and HbA1c (model 1). Further adjustments were made for LDL, HDL, systolic blood pressure, eGFR, antihypertensive medication, triglycerides, BMI and smoking, and for the presence of CVD, albuminuria and/or retinopathy, as appropriate (model 2). Markers of ED (model 3) and LGI (model 4) were subsequently added to this model to explore whether the association between plasma levels of MMPs and TIMP-1 on the one hand and CVD and microvascular complications on the other (if any) were explained (i.e., potentially mediated) by LGI and/or ED. Markers of LGI were comprised into an overall LGI z-score by computing a z-score after averaging the z-scores of lnCRP, lnIL-6 and lnTNF-α. ED z-scores were expressed after averaging of the z-scores of sVCAM-1 and sE-selectin. A z-score represents the difference between the individual biomarker score and the mean value in the population, expressed in units of the standard deviation of the study population. This enables comparison of markers expressed in different units. The two scores represent a more robust value of the individual’s levels of LGI and ED, as they reduce the influence of biological variability expected when LGI and ED should be characterized by levels of each biomarker separately [25].

## Results

### Patients’ characteristics

Table 1 shows the characteristics of patients with or without vascular complications. Patients with vascular complications were characterized by higher age, BMI and systolic blood pressure. Levels of HbA1c, LDL, triglycerides, MMP-1, MMP-2, MMP-3, MMP-9, MMP-10, TIMP-1 and markers of LGI and ED were significantly higher in individuals with vascular complications compared to those without.

**Table 1:** **Clinical characteristics of the study population**

	**Vascular complications**	**No vascular complications**	**p-value**
	**(n = 306)**	**(n = 187)**	
Age (years)	41.6 (10.4)	36.1 (8.1)	<0.001
Sex (male/female, %)	54/46	46/54	0.096
BMI (kg/m^2^)	24.8 (3.5)	23.9 (2.6)	<0.001
HbA1c (%)	9.0 (1.6)	7.7 (1.3)	<0.001
HbA1c (mmol/mol)	75 (17.4)	61 (14.1)	<0.001
Duration of diabetes (years)	25.2 (8.9)	15.5 (7.0)	<0.001
LDL cholesterol (mmol/l)	3.30 (1.06)	2.88 (0.93)	<0.001
HDL cholesterol (mmol/l)	1.59 (0.42)	1.68 (0.45)	0.032
Triglycerides (mmol/l)	1.13 [0.84-1.58]	0.85 [0.67-1.09]	<0.001
Smoking (no/former/current) (%)	36/31/33	46/26/28	0.047
Systolic blood pressure (mmHg)	127 (21)	115 (13)	<0.001
Diastolic blood pressure (mmHg)	75 (12)	73 (10)	0.085
Serum creatinine (μmol/l)	76.0 [68.0-90.0]	71.0 [64.0-79.0]	<0.001
eGFR (ml/min/1.73 m^2^)	95.7 [79.0-107.9]	107.9 [97.2-115.6]	<0.001
Cardiovascular disease (%)	38.6	-	-
Albuminuria (normo-/micro-/macro-)(%)	37.9/25.2/36.9	-	-
Retinopathy (no/background/proliferative) (%)	11.4/40.9/47.7	-	-
Antihypertensive medication (%)	45.4	5.9	<0.001
ACE-inhibitor (%)	37.6	4.3	<0.001
MMP-1 (ng/ml)	12.8 [7.0-19.9]	10.8 [5.9-16.5]	0.006
MMP-2 (ng/ml)	110 [100–120]	103 [96–110]	<0.001
MMP-3 (ng/ml)	17.8 [11.2-28.3]	12.9 [8.3-19.6]	<0.001
MMP-9 (ng/ml)	122 [81–195]	110 [66–172]	0.023
MMP-10 (pg/ml)	1285 [942–1931]	1077 [790–1635]	<0.001
TIMP-1 (ng/ml)	313 (100)	256 (74)	<0.001
C-reactive protein (mg/l)	1.28 [0.46-2.68]	0.71 [0.35-1.80]	<0.001
Interleukin-6 (pg/ml)	2.12 [1.35-3.86]	1.57 [1.06-2.50]	<0.001
Tumor necrosis factor-α (pg/ml)	3.16 [2.33-4.42]	2.22 [1.68-2.85]	<0.001
Soluble e-selectin (ng/ml)	35.7 (16.6)	31.1 (11.1)	<0.001
Soluble vascular cell adhesion molecule-1 (ng/ml)	435 (144)	378 (104)	<0.001

Data are presented as means (standard deviation), median [inter-quartile range], or percentages, as appropriate.

Vascular complication: presence of previous CVD, macroalbuminuria, or proliferative retinopathy or the combination of microalbuminuria and non-proliferative retinopathy; BMI, body mass index; HbA1c, glycated hemoglobin; LDL, low-density lipoprotein; HDL, high-density lipoprotein; eGFR, estimated glomerular filtration rate by CKD-EPI formula; MMP, matrix metalloproteinase; TIMP-1, tissue inhibitor of metalloproteinase-1.

### Associations between MMPs, TIMP-1 and cardiovascular disease

**Figure 1:**
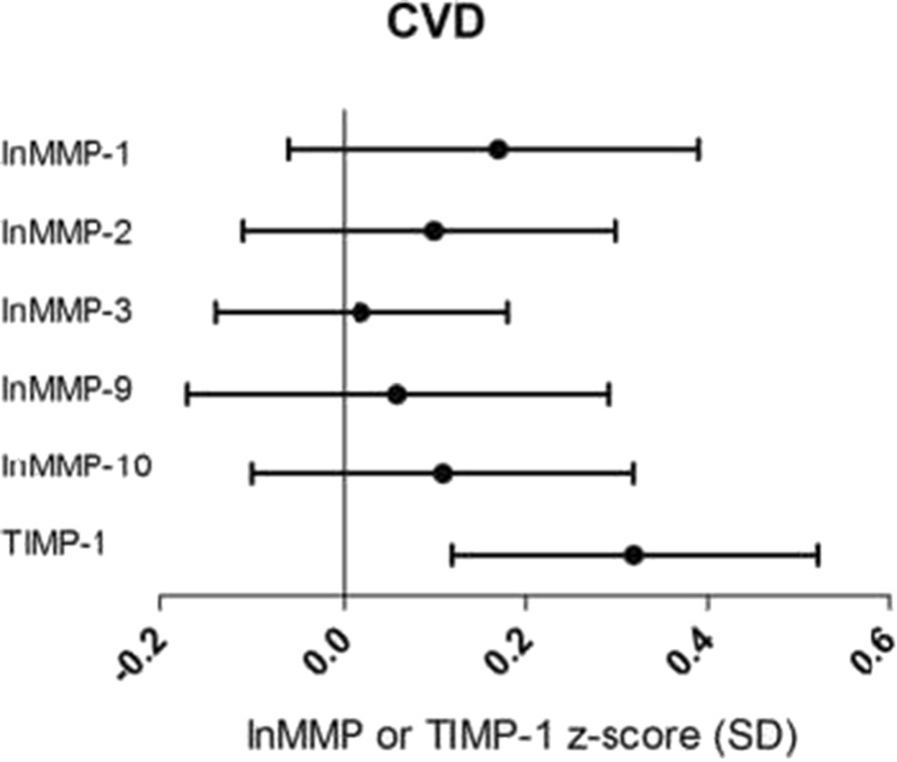
**Associations between plasma levels of MMPs, TIMP-1 and CVD.** Point estimates and 95% confidence intervals show the difference in plasma levels of lnMMP or TIMP-1 (in SD) in patients with vs. those without CVD resulting from a multivariable regression model including all cardiovascular risk factors, albuminuria and retinopathy (model 2).

Significantly higher plasma levels of TIMP-1 [β = 0.27 SD (95%CI: 0.06; 0.48)] were observed in individuals with CVD (n = 118) as compared to those without, after adjustment for age, sex, duration of diabetes and HbA1c (Additional file 1: Table S1, Model 1). The association became even stronger [0.32 (0.12; 0.52)] after further adjustment for other cardiovascular risk factors, albuminuria and retinopathy (Figure 1; Additional file 1: Table S1, Model 2). In contrast, MMP-1, MMP-2, MMP-3, MMP-9 and MMP-10 did not differ between groups.

### Associations between MMPs, TIMP-1 and albuminuria

**Figure 2:**
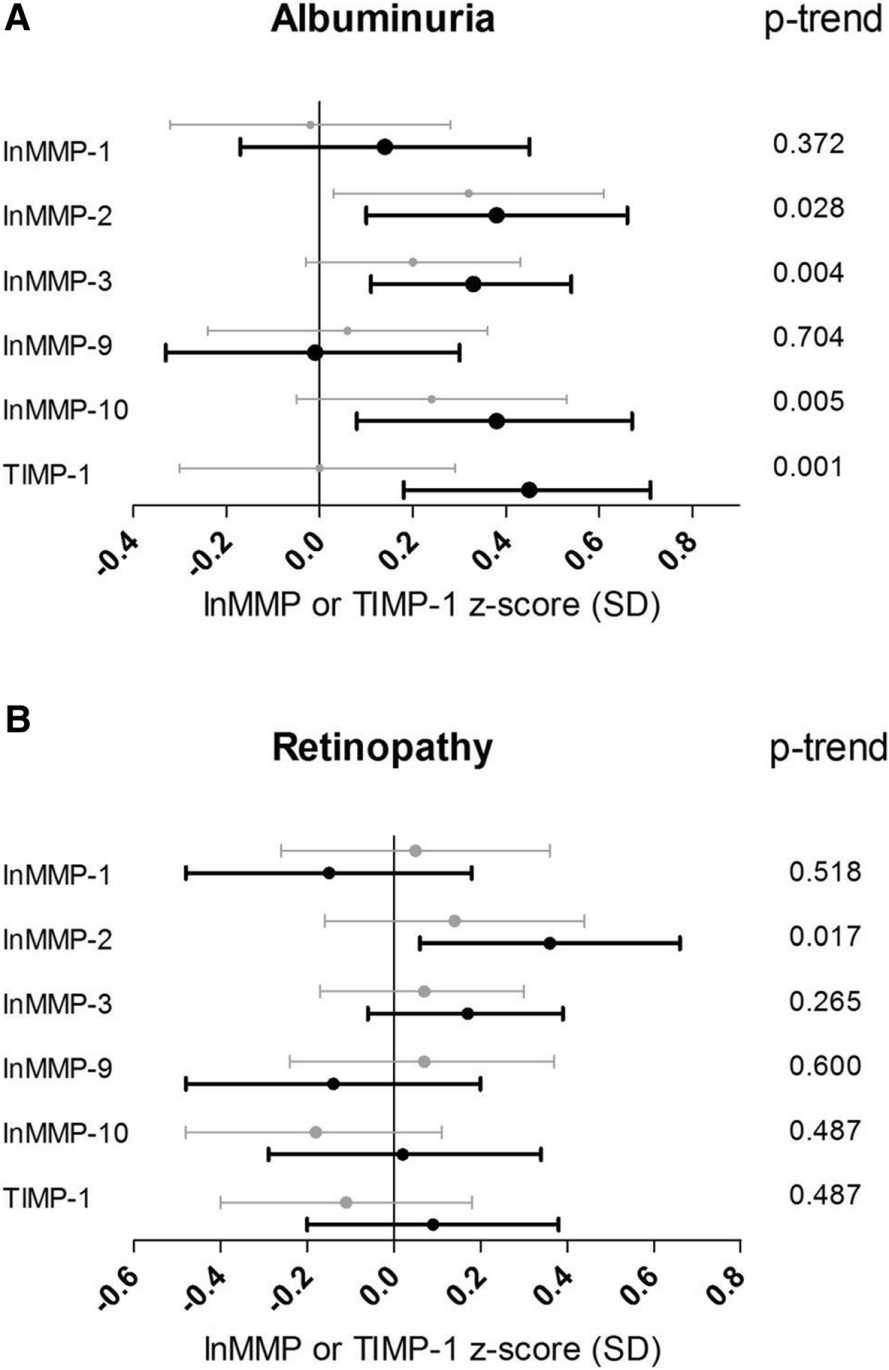
**Associations between plasma levels of MMPs, TIMP-1 and microvascular complications.** Point estimates and 95% confidence intervals show the difference in plasma levels of lnMMP or TIMP-1 (in SD) in patients with vs. those without microvascular complications resulting from a multivariable regression model including all cardiovascular risk factors and the other vascular complications (model 2). **A**, differences in micro- (grey bars) or macroalbuminuria (black bars) compared to normoalbuminuria; **B**, differences in non-proliferative (grey bars) and proliferative retinopathy (black bars) compared to no retinopathy. P-trend indicates the statistical significance of the associations between plasma levels of MMPs or TIMP and the degrees of albuminuria (normo- vs. micro- vs. macroalbuminuria) or retinopathy (no vs. non-proliferative vs. proliferative retinopathy).

Patients with microalbuminuria (n = 77) showed significantly higher plasma levels of MMP-2 compared to patients with normal urinary albumin excretion after adjustment for cardiovascular risk factors and other vascular complications [0.32 (0.03; 0.61)] (Figure 2A; Additional file 2: Table S2, Model 2). Macroalbuminuria (n = 113), compared to normoalbuminuria, was associated with even higher levels of MMP-2 [0.38 (0.10; 0.66)] (Figure 2A; Additional file 2: Table S2). In this group, additionally, higher plasma levels of MMP-3 [0.33 (0.11; 0.54)], MMP-10 [0.38 (0.08; 0.67)] and TIMP-1 [0.45 (0.18; 0.71)] were found. Significant associations with the degree of albuminuria (normo- vs. micro- vs. macroalbuminuria) were observed for higher plasma levels of MMP-2 (p-trend = 0.028), MMP-3 (p-trend = 0.004), MMP-10 (p-trend = 0.005) and TIMP-1 (p-trend = 0.001) (Figure 2A).

### Associations between MMPs, TIMP-1 and retinopathy

Proliferative retinopathy (n = 146) was associated with higher levels of MMP-2 [0.71 (0.45; 0.96)], MMP-3 [0.49 (0.28; 0.70)], MMP-10 [0.39 (0.11; 0.66)] and TIMP-1 [0.54 (0.28; 0.81)] after adjustment for age, sex, duration of diabetes and HbA1c (Additional file 3: Table S3, Model 1). However, after further adjustment for the other cardiovascular risk factors, CVD and albuminuria, only MMP-2 [0.36 (0.06; 0.66)] remained significantly higher in patients with proliferative retinopathy vs. those without retinopathy (Figure 2B; Additional file 3: Table S3, Model 2). In addition, a significant trend across the severity of retinopathy was observed for increasing plasma levels of MMP-2 (p-trend = 0.017).

In contrast, non-proliferative retinopathy (n = 125) was not significantly associated with higher plasma levels of MMPs or TIMP-1 (Figure 2B; Additional file 3: Table S3).

### Associations of MMPs and TIMP-1 with LGI and ED

Associations between MMPs and TIMP-1 and the individual markers of LGI and ED are shown in Additional file 4: Table S4. After adjustment for all cardiovascular risk factors (model 2), MMP-1, −3, −9, −10, and TIMP-1, but not MMP-2, were positively and significantly associated with the LGI score. In addition, MMP-2, MMP-10 and TIMP-1 were positively and significantly associated with the ED score (Table 2, model 2). However, significant associations between plasma levels of MMPs and TIMP-1 on the one hand and CVD, albuminuria and retinopathy on the other hand were largely independent of LGI and ED, as these associations remained significant after adding the LGI and/or ED score to the model (Additional file 1: Table S1, Additional file 2: Table S2 and Additional file 3: Table S3; Models 3, 4 and 5).

**Table 2:** **Associations between lnMMP-1, −2, −3, −9, −10 and TIMP-1 and the LGI and ED scores**

		**Inflammation (z-score)**			**Endothelial dysfunction (z-score)**		
	**Model**	**β**	**95% CI**	**p-value**	**β**	**95% CI**	**p-value**
**MMP-1**	1	0.07	−0.02;0.15	0.112	0.00	−0.09;0.09	0.998
	2	0.08	0.00;0.16	0.047	0.00	−0.09;0.08	0.987
	3	0.08	−0.00;0.15	0.062	−0.01	−0.09;0.08	0.834
**MMP-2**	1	0.10	0.01;0.18	0.032	0.19	0.10;0.27	<0.001
	2	0.03	−0.06;0.12	0.526	0.14	0.04;0.23	0.004
	3	0.00	−0.08;0.09	0.950	0.13	0.04;0.22	0.006
**MMP-3**	1	0.26	0.16;0.36	<0.001	0.08	−0.03;0.19	0.137
	2	0.19	0.09;0.30	0.001	−0.02	−0.14;0.10	0.715
	3	0.17	0.06;0.28	0.003	−0.04	−0.16;0.08	0.474
**MMP-9**	1	0.16	0.08;0.25	<0.001	−0.02	−0.11;0.06	0.592
	2	0.17	0.09;0.24	<0.001	−0.02	−0.10;0.07	0.718
	3	0.16	0.08;0.24	<0.001	−0.02	−0.10;0.06	0.577
**MMP-10**	1	0.24	0.16;0.32	<0.001	0.13	0.04;0.21	0.003
	2	0.22	0.14;0.30	<0.001	0.11	0.02;0.20	0.019
	3	0.21	0.13;0.30	<0.001	0.10	0.01;0.19	0.029
**TIMP-1**	1	0.34	0.27;0.43	<0.001	0.25	0.17;0.33	<0.001
	2	0.29	0.21;0.38	<0.001	0.21	0.12;0.30	<0.001
	3	0.28	0.20;0.37	<0.001	0.20	0.11;0.29	<0.001

β, standardized regression coefficient: indicates increase in the low-grade inflammation and endothelial dysfunction score (in SDs) per 1 SD increase in lnMMP-1, −2, −3, −9, −10 or TIMP-1. CI: confidence interval. MMP: matrix metalloproteinase.

Model 1: adjusted for age, sex, duration of diabetes and HbA1c.

Model 2: model 1 + BMI, triglycerides, LDL, HDL, systolic blood pressure, eGFR, smoking and antihypertensive medication.

Model 3: model 2 + CVD, albuminuria and retinopathy.

### Additional analyses

Adding use of ACE inhibitors specifically versus antihypertensive medication in general as covariable to our models, did not materially influence the results. Similarly, adjustment for urinary albumin excretion rate instead of albuminuria status and waist-hip-ratio instead of BMI did not change our results (data not shown). We have not included adjustment for TIMP-1 in model 2 in the main analyses between plasma levels of MMPs and vascular complications, because TIMP-1 levels are influenced by all MMPs and not just by one MMP. Therefore, we have also not analyzed associations between MMP/TIMP-1 ratios and vascular complications. However, additional adjustment in model 2 for TIMP-1 levels did also not materially influence our results.

In the relationship between MMPs, TIMP and vascular complications, eGFR (a marker of renal dysfunction) can be considered as a potential confounder or mediator. If eGFR should be a mediator rather than a confounder, models including eGFR may be overadjusted. We therefore reanalyzed the data without adjustment for eGFR; results were generally similar to models with such adjustment (data not shown).

## Discussion

This is the first study that comprehensively assessed associations of MMPs and TIMP-1 with long-term complications in type 1 diabetes. In a group of 493 patients with and without diabetes complications, we found that high plasma levels of TIMP-1 were associated with CVD. In addition, MMP-2, -3 and -10, and TIMP-1 levels were associated with macroalbuminuria, and MMP-2 was associated with proliferative retinopathy. All these associations remained significant after adjustment for cardiovascular risk factors, prevalence of other vascular complications and markers of LGI and ED.

### MMPs, TIMP-1 and CVD

Prevalent CVD was associated with higher plasma levels of TIMP-1, but not of MMP-1, −2, −3, −9 and −10. In accordance, significantly higher levels of plasma TIMP-1 have been previously observed in two studies in type 2 diabetic patients with a history of coronary artery disease [26,27]. In addition, in a large prospective cohort study of individuals with and without (presumably type 2) diabetes, increased plasma TIMP-1 levels, measured six months after myocardial infarction, was significantly associated with left ventricular remodeling and adverse outcomes [28]. In the current study on individuals with type 1 diabetes, measurements of plasma TIMP-1 were, by its cross-sectional design, performed 1 month to 10 years after the cardiovascular event. Although we cannot prove a relationship between elevated plasma levels of TIMP-1 and new cardiovascular events, TIMP-1 may be a marker for abnormal regulation of matrix remodeling after a cardiovascular event. This is supported by studies in animal models showing elevation of plasma TIMP-1 in type 1 diabetic minipigs with left ventricular hypertrophy, increased cardiac fibrosis and cardiac dysfunction [29].

### MMPs, TIMP-1 and albuminuria

Plasma levels of MMP-2 were significantly higher in both micro- and macroalbuminuric patients compared to those with normoalbuminuria. In contrast, a previous study in type 1 diabetic patients did not show increased levels of MMP-2 in microalbuminuric vs. normoalbuminuric individuals [7]. This discrepancy may be explained by the very small sample size (n = 12) of that study [7].

Thus far, no studies have been reported regarding plasma MMP-3 and diabetic nephropathy in humans or animals. Our study is the first study showing that higher plasma levels of MMP-3 are associated with increasing levels of albuminuria. In accordance with our study, in type 2 diabetic patients, high interstitial MMP-3 mRNA expression was associated with higher degrees of interstitial injury [30]. The increased plasma MMP-3 could therefore potentially originate from the renal interstitium. This finding may be biologically explained by the fact that type IV collagen, one of the major substrates of proteolysis by MMP-3, is an important component of the basal lamina of the glomerular basement membrane, Bowman’s capsule, and of the basal membranes of renal tubules and vessels [31]. MMP-3-dependent type IV collagen proteolysis therefore constitutes a potential explanation for the current observation of a strong association between MMP-3 and albuminuria.

In addition, higher plasma MMP-10 levels were significantly associated with macroalbuminuria in our study. This result is in full agreement with a recent study [8] in patients with type 1 diabetes, in which a higher plasma level of MMP-10 was associated with diabetic nephropathy independently of classical risk factors. In addition, MMP-10 knockout (streptozotocin (STZ)-induced) diabetic mice showed lower renal mesangial cell expansion and lower renal macrophage influx compared to wildtype diabetic mice [8], suggesting that MMP-10 may be involved in the onset of diabetic nephropathy.

Besides MMP-2, -3, and -10, TIMP-1 was also associated with macroalbuminuria. This association was also observed in one-sided nephrectomized type 1 diabetic rats compared to non-diabetic controls [32]. These results reflect a possible role for TIMP-1 in increased renal fibrosis in early diabetic nephropathy.

### MMPs, TIMP-1 and retinopathy

Plasma MMP-2 levels showed a significant positive association with proliferative retinopathy. Salzmann *et al.* [33] showed an overall increase of different MMPs (−2, −3 and −9) and TIMP-1 in specimens of retinal tissue of patients with proliferative diabetic retinopathy compared to retinal tissue of patients without diabetes. Also, increased retinal levels of MMP-2 were found in rats and mice with STZ-induced diabetes compared to non-diabetic controls [34]. Our current clinical results, supported by the relatively scarce literature, indicate that MMP-2 may play a role the pathogenesis of proliferative diabetic retinopathy. In addition, the study by Toni *et al.* [8] has shown a significant association between plasma MMP-10 and proliferative retinopathy, which is in accordance with our current findings in the minimally adjusted model (including age, sex, duration of diabetes and HbA1c). However, this was no longer significant after additional adjustment for cardiovascular risk factors and the presence of CVD and albuminuria. Since Toni *et al*. did not adjust for the presence of other vascular complications, this could explain the observed difference between their and our study.

We did not find a significant association of plasma MMPs or TIMP-1 levels with non-proliferative retinopathy. This is in contrast with the results of the study of Jacqueminet *et al.* [10], in which increased serum MMP-9 levels were shown in patients with type 1 diabetes with minimal to moderate retinopathy (n = 14) compared to diabetic patients without retinopathy and non-diabetic controls. Possibly the serum measurement does not truly reflect the circulating concentration of MMPs and TIMPs compared to the plasma measurement [35]. In addition, potential confounders may not have been fully addressed in this earlier study, but were shown to be of importance in the current study. In fact, also the current study showed a significant association between plasma MMP-9 levels and the presence of non-proliferative retinopathy in the crude model [standardized β = 0.22 (0.00;0.44)], but this was attenuated after adjustment for confounding.

### MMPs, TIMP-1, low-grade inflammation (LGI) and endothelial dysfunction (ED)

In our study, all MMPs, except MMP-2, were significantly associated with markers of low-grade inflammation (Table 2, model 2). Indeed, it is well known that MMP-2 has a more potent anti-inflammatory effect compared to other MMPs, which may be attributable to the fact that monocyte chemo-attractant protein-3 (MCP-3) is an important substrate of MMP-2. The product of MCP-3 after cleavage by MMP-2 antagonizes the effect of MCP-3 on the chemokine receptors-1, −2 and −3 and may thereby attenuate the inflammatory response [36]. Although MMP-1 and MMP-3 are known to cleave MCP-1, −2 and −4, the antagonistic effects of the cleaved products on inflammation are minimal, because of lower affinity for the chemokine receptors [37]. MMPs can be activated and up-regulated by mediators of inflammation, but also have an intrinsic effect on the inflammatory response [38]. Although the exact role of MMPs in the inflammatory cascade remains incompletely understood, our results suggest that associations of MMPs and TIMP-1 with complications of type 1 diabetes are unlikely to be explained by LGI.

MMP-2, MMP-10 and TIMP-1 were associated with markers of ED. These findings are in line with literature demonstrating that MMP-2 is able to degrade occludin, a tight junction protein, in the blood retinal membrane leading to increased vascular permeability [12], and that MMP-10 can induce endothelial proteolysis in response to CRP [39]. In addition, plasma TIMP-1 levels are associated with endothelial cell migration [40]. Thus, associations between MMP and TIMP-1 levels and ED are biologically plausible; nevertheless, our results suggest that associations of MMPs and TIMP-1 with complications of type 1 diabetes are unlikely to be explained by ED.

### Limitations

There are several limitations to our study. First, the cross-sectional design only allows speculations on causality. We thus cannot exclude that increased levels of plasma MMPs and TIMP-1 may be a result instead of a cause of vascular complications. Second, circulating levels of biomarkers may reflect specific organ injury, but can also originate from other tissues. We adjusted for other known vascular complications, but cannot rule out that other causative factors may contribute and the observed associations could therefore be underestimated. In addition, MMP-1, −3, −10 and TIMP-1 have a smaller molecular weight than albumin [41,42] and may be filtered in the glomerulus, especially in albuminuric patients, which may also have underestimated the associations between these biomarkers and vascular complications. Third, plasma samples can be obtained easily and we thus used plasma levels to investigate the involvement of MMPs and TIMP-1 in the pathogenesis of vascular complications in type 1 diabetes. However, we do not know whether plasma markers truly reflect the local pathological situation at the tissue level. Finally, our results suggest that associations of MMPs and TIMP-1 with complications of type 1 diabetes are unlikely to be explained by LGI or ED, but we cannot exclude roles of LGI or ED not reflected by the biomarkers we used.

## Conclusion

In this study with type 1 diabetic patients, plasma MMP-2, MMP-3, MMP-10 and TIMP-1 levels are associated with macro- and microvascular complications and these associations were largely independent of LGI and ED. Further (prospective) studies are warranted to elucidate the involvement of MMPs and TIMPs in the development of diabetic macro- and microvascular complications.

## Additional files

Additional file 1: Table S1. Associations between lnMMP-1, lnMMP2, lnMMP-3, lnMMP-9, lnMMP-10 and TIMP-1 and cardiovascular disease. Additional file 2: Table S2. Associations between lnMMP-1, lnMMP-2, lnMMP-3, lnMMP-9, lnMMP-10 and TIMP-1 and microalbuminuria or macroalbuminuria. Additional file 3: Table S3. Associations between lnMMP-1, lnMMP-2, lnMMP-3, lnMMP-9, lnMMP-10 and TIMP-1 and non-proliferative or proliferative retinopathy. Additional file 4: Table S4. Associations between plasma levels of MMP-1, -2, -3, -9, and -10 and TIMP-1 and markers of low-grade inflammation and endothelial dysfunction.

## Abbreviations

CVD: Cardiovascular disease
ED: Endothelial dysfunction
LGI: Low-grade inflammation
MMP: Matrix metalloproteinase
TIMP: Tissue inhibitor of metalloproteinase
