# Erratum to: Plasma levels of matrix metalloproteinase-2, -3, -10, and tissue inhibitor of metalloproteinase-1 are associated with vascular complications in patients with type 1 diabetes: the EURODIAB Prospective Complications Study

**DOI:** 10.1186/s12933-015-0290-4

**Published:** 2015-09-28

**Authors:** Stijn A Peeters, Lian Engelen, Jacqueline Buijs, Nish Chaturvedi, John H Fuller, Casper G Schalkwijk, Coen D Stehouwer, EURODIAB Prospective Complications Study Group

**Affiliations:** Department of Internal Medicine, Maastricht University Medical Centre, P.O. Box 5800, 6202 AZ Maastricht, the Netherlands; Department of Internal Medicine, Atrium Medical Centre, Heerlen, the Netherlands; Institute of Cardiovascular Sciences, University College London, London, UK; Department of Epidemiology and Public Health, University College London, London, UK

## Erratum to: Cardiovascular Diabetology (2015) 14:31 DOI 10.1186/s12933-015-0195-2

After publication of the original article, it came to our attention that the samples used for the MMPs and TIMP-1 measurements were serum samples instead of plasma samples. Therefore,

1. *Plasma* levels of MMPs and TIMP-1 in the title, abstract, methods, results and discussion section should read *serum* levels; and
2. In the discussion section the sentence, “Possibly the serum measurement does not truly reflect the circulating concentration of MMPs and TIMPs compared to the plasma measurement” should be disregarded.

We apologize for any inconvenience.

