# Supplementary material for: Plasma levels of matrix metalloproteinase-2, -3, -10, and tissue inhibitor of metalloproteinase-1 are associated with vascular complications in patients with type 1 diabetes: the EURODIAB Prospective Complications Study
Source: Cardiovasc Diabetol. 2015 Mar 10;14:31. doi: 10.1186/s12933-015-0195-2 (PMC4355971; doi:10.1186/s12933-015-0195-2)
Supplement: Additional file 1: Table S1. — Associations between lnMMP-1, lnMMP2, lnMMP-3, lnMMP-9, lnMMP-10 and TIMP-1 and cardiovascular disease. [file 12933_2015_195_MOESM1_ESM.doc]

| **Additional table S1. Associations between lnMMP-1, lnMMP2, lnMMP-3, lnMMP-9, lnMMP-10 and TIMP-1 and cardiovascular disease .** | | | | | | | | | | | | | | | | | | | | | | | |
| --- | --- | --- | --- | --- | --- | --- | --- | --- | --- | --- | --- | --- | --- | --- | --- | --- | --- | --- | --- | --- | --- | --- | --- |
|  |  | lnMMP-1 |  |  |  | lnMMP-2 |  |  |  | lnMMP-3 |  |  |  | lnMMP-9 |  |  |  | lnMMP-10 |  |  |  | TIMP-1 |  |
| Model | β | 95% CI | p-value |  | β | 95% CI | p-value |  | β | 95% CI | p-value |  | β | 95% CI | p-value |  | β | 95% CI | p-value |  | β | 95% CI | p-value |
| 1 | 0.16 | -0.06;0.37 | 0.161 |  | 0.11 | -0.10;0.32 | 0.320 |  | 0.03 | -0.15;0.20 | 0.746 |  | 0.06 | -0.16;0.28 | 0.567 |  | 0.07 | -0.15;0.29 | 0.544 |  | **0.27** | **0.06;0.48** | **0.012** |
| 2 | 0.17 | -0.06;0.39 | 0.147 |  | 0.10 | -0.11;0.30 | 0.352 |  | 0.02 | -0.14;0.18 | 0.792 |  | 0.06 | -0.17;0.29 | 0.588 |  | 0.11 | -0.10;0.32 | 0.315 |  | **0.32** | **0.12;0.52** | **0.002** |
| 3 | 0.17 | -0.06;0.39 | 0.145 |  | 0.08 | -0.12;0.29 | 0.419 |  | 0.02 | -0.14;0.19 | 0.767 |  | 0.07 | -0.16;0.29 | 0.571 |  | 0.10 | -0.11;0.31 | 0.365 |  | **0.30** | **0.11;0.50** | **0.003** |
| 4 | 0.16 | -0.07;0.38 | 0.173 |  | 0.10 | -0.11;0.30 | 0.354 |  | 0.01 | -0.15;0.17 | 0.905 |  | 0.04 | -0.18;0.26 | 0.726 |  | 0.08 | -0.12;0.29 | 0.436 |  | **0.29** | **0.10;0.48** | **0.003** |
| 5 | 0.16 | -0.07;0.38 | 0.165 |  | 0.09 | -0.12;0.29 | 0.401 |  | 0.01 | -0.14;0.17 | 0.859 |  | 0.05 | -0.18;0.27 | 0.677 |  | 0.08 | -0.13;0.29 | 0.448 |  | **0.28** | **0.09;0.47** | **0.004** |

The standardized regression coefficient β represents the difference in plasma levels of MMPs and TIMP-1 (in SD) in patients with (n=118) vs. those without cardiovascular disease (n=375).

| model 1 | adjusted for age, sex, duration of diabetes and HbA1c | | |  |  |
| --- | --- | --- | --- | --- | --- |
| model 2 | model 1 + BMI, LDL, HDL, triglycerides, systolic blood pressure, eGFR, smoking, antihypertensive medication, albuminuria and retinopathy | | | | |
| model 3 | model 2 + endothelial dysfunction score | |  |  |  |
| model 4 | model 2 + inflammation score |  |  |  |  |
| model 5 | model 2 + endothelial score and inflammation score | | | | |
