# Supplementary material for: Plasma levels of matrix metalloproteinase-2, -3, -10, and tissue inhibitor of metalloproteinase-1 are associated with vascular complications in patients with type 1 diabetes: the EURODIAB Prospective Complications Study
Source: Cardiovasc Diabetol. 2015 Mar 10;14:31. doi: 10.1186/s12933-015-0195-2 (PMC4355971; doi:10.1186/s12933-015-0195-2)
Supplement: Additional file 2: Table S2. — Associations between lnMMP-1, lnMMP-2, lnMMP-3, lnMMP-9, lnMMP-10 and TIMP-1 and microalbuminuria or macroalbuminuria. [file 12933_2015_195_MOESM2_ESM.doc]

| **Additional table S2. Associations between lnMMP-1, lnMMP-2, lnMMP-3, lnMMP-9, lnMMP-10 and TIMP-1 and microalbuminuria or macroalbuminuria.** | | | | | | | | | | | | | | | | | | | | | | |  | |
| --- | --- | --- | --- | --- | --- | --- | --- | --- | --- | --- | --- | --- | --- | --- | --- | --- | --- | --- | --- | --- | --- | --- | --- | --- |
| Microalbuminuria | | |  |  |  |  |  |  |  |  |  |  |  |  |  |  |  |  |  |  |  |  | |  |
|  |  | lnMMP-1 |  |  |  | lnMMP-2 |  |  |  | lnMMP-3 |  |  |  | lnMMP-9 |  |  |  | lnMMP-10 |  |  |  | TIMP-1 | |  |
| Model | β | 95% CI | p-value |  | β | 95% CI | p-value |  | β | 95% CI | p-value |  | β | 95% CI | p-value |  | β | 95% CI | p-value |  | β | 95% CI | | p-value |
| 1 | -0.02 | -0.29;0.25 | 0.875 |  | **0.44** | **0.17;0.70** | **0.001** |  | **0.29** | **0.08;0.51** | **0.008** |  | 0.11 | -0.16;0.39 | 0.420 |  | 0.27 | 0.00;0.54 | 0.052 |  | 0.15 | -0.13;0.42 | | 0.291 |
| 2 | -0.02 | -0.32;0.28 | 0.917 |  | **0.32** | **0.03;0.61** | **0.028** |  | 0.20 | -0.03;0.43 | 0.087 |  | 0.06 | -0.24;0.36 | 0.701 |  | 0.24 | -0.05;0.53 | 0.109 |  | 0.00 | -0.30;0.29 | | 0.979 |
| 3 | -0.01 | -0.31;0.29 | 0.933 |  | 0.28 | -0.01;0.57 | 0.057 |  | 0.20 | -0.03;0.44 | 0.084 |  | 0.07 | -0.24;0.38 | 0.650 |  | 0.21 | -0.08;0.50 | 0.150 |  | -0.05 | -0.35;0.24 | | 0.713 |
| 4 | -0.03 | -0.33;0.27 | 0.831 |  | **0.33** | **0.04;0.62** | **0.028** |  | 0.18 | -0.05;0.41 | 0.127 |  | 0.01 | -0.29;0.31 | 0.955 |  | 0.18 | -0.11;0.46 | 0.220 |  | -0.07 | -0.36;0.21 | | 0.620 |
| 5 | -0.03 | -0.33;0.28 | 0.866 |  | **0.29** | **0.00;0.58** | **0.048** |  | 0.19 | -0.04;0.42 | 0.111 |  | 0.03 | -0.27;0.33 | 0.842 |  | 0.17 | -0.11;0.46 | 0.230 |  | -0.09 | -0.38;0.19 | | 0.517 |
|  |  |  |  |  |  |  |  |  |  |  |  |  |  |  |  |  |  |  |  |  |  |  | |  |
| Macroalbuminuria | | |  |  |  |  |  |  |  |  |  |  |  |  |  |  |  |  |  |  |  |  | |  |
|  |  | lnMMP-1 |  |  |  | lnMMP-2 |  |  |  | lnMMP-3 |  |  |  | lnMMP-9 |  |  |  | lnMMP-10 |  |  |  | TIMP-1 | |  |
| Model | β | 95% CI | p-value |  | β | 95% CI | p-value |  | β | 95% CI | p-value |  | β | 95% CI | p-value |  | β | 95% CI | p-value |  | β | 95% CI | | p-value |
| 1 | 0.16 | -0.09;0.41 | 0.218 |  | **0.60** | **0.37;0.83** | **<0.001** |  | **0.59** | **0.41;0.78** | **<0.001** |  | 0.06 | -0.19;0.31 | 0.650 |  | **0.52** | **0.27;0.76** | **<0.001** |  | **0.76** | **0.53;0.99** | | **<0.001** |
| 2 | 0.14 | -0.17;0.45 | 0.391 |  | **0.38** | **0.10;0.66** | **0.009** |  | **0.33** | **0.11;0.54** | **0.003** |  | -0.01 | -0.33;0.30 | 0.934 |  | **0.38** | **0.08;0.67** | **0.012** |  | **0.45** | **0.18;0.71** | | **0.001** |
| 3 | 0.14 | -0.18;0.45 | 0.394 |  | **0.35** | **0.07;0.63** | **0.015** |  | **0.33** | **0.11;0.55** | **0.004** |  | -0.01 | -0.33;0.31 | 0.952 |  | **0.33** | **0.04;0.62** | **0.028** |  | **0.39** | **0.13;0.66** | | **0.004** |
| 4 | 0.11 | -0.20;0.42 | 0.478 |  | **0.37** | **0.08;0.65** | **0.011** |  | **0.29** | **0.08;0.51** | **0.008** |  | -0.09 | -0.40;0.23 | 0.590 |  | 0.29 | 0.00;0.57 | 0.050 |  | **0.36** | **0.10;0.62** | | **0.007** |
| 5 | 0.12 | -0.19;0.43 | 0.456 |  | **0.35** | **0.06;0.63** | **0.016** |  | **0.30** | **0.08;0.52** | **0.007** |  | -0.06 | -0.38;0.25 | 0.689 |  | 0.27 | -0.01;0.56 | 0.062 |  | **0.34** | **0.08;0.60** | | **0.011** |

The standardized regression coefficient β represents the difference in plasma levels of MMPs and TIMP-1 (in SD) in patients with microalbuminuria (n=77) or macroalbuminuria (n=113) vs. those with normoalbuminuria (n=303).

| model 1 | adjusted for age, sex, duration of diabetes and HbA1c | |  | |
| --- | --- | --- | --- | --- |
| model 2 | model 1 + BMI, LDL, HDL, triglycerides, systolic blood pressure, eGFR, smoking, antihypertensive medication, CVD and retinopathy | | | |
| model 3 | model 2 + endothelial dysfunction score |  | | |
| model 4 | model 2 + inflammation score |  | | |
| model 5 | model 2 + endothelial score and inflammation score | | |  |
