# Supplementary material for: Plasma levels of matrix metalloproteinase-2, -3, -10, and tissue inhibitor of metalloproteinase-1 are associated with vascular complications in patients with type 1 diabetes: the EURODIAB Prospective Complications Study
Source: Cardiovasc Diabetol. 2015 Mar 10;14:31. doi: 10.1186/s12933-015-0195-2 (PMC4355971; doi:10.1186/s12933-015-0195-2)
Supplement: Additional file 3: Table S3. — Associations between lnMMP-1, lnMMP-2, lnMMP-3, lnMMP-9, lnMMP-10 and TIMP-1 and non-proliferative or proliferative retinopathy. [file 12933_2015_195_MOESM3_ESM.doc]

**Additional table S3 Associations between lnMMP-1, lnMMP-2, lnMMP-3, lnMMP-9, lnMMP-10 and TIMP-1 and non-proliferative or proliferative retinopathy.**

| Non-proliferative retinopathy | | | |  |  |  |  |  |  |  |  |  |  |  |  |  |  |  |  |  |  |  |  |
| --- | --- | --- | --- | --- | --- | --- | --- | --- | --- | --- | --- | --- | --- | --- | --- | --- | --- | --- | --- | --- | --- | --- | --- |
|  |  | lnMMP-1 |  |  |  | lnMMP-2 |  |  |  | lnMMP-3 |  |  |  | lnMMP-9 |  |  |  | lnMMP-10 |  |  |  | TIMP-1 |  |
| Model | β | 95% CI | p-value |  | β | 95% CI | p-value |  | β | 95% CI | p-value |  | β | 95% CI | p-value |  | β | 95% CI | p-value |  | β | 95% CI | p-value |
| 1 | 0.06 | -0.20;0.31 | 0.661 |  | 0.22 | -0.03;0.47 | 0.085 |  | 0.19 | -0.02;0.40 | 0.069 |  | 0.17 | -0.09;0.42 | 0.202 |  | -0.01 | -0.27;0.24 | 0.930 |  | 0.17 | -0.08;0.43 | 0.179 |
| 2 | 0.05 | -0.26;0.36 | 0.750 |  | 0.14 | -0.16;0.44 | 0.354 |  | 0.07 | -0.17;0.30 | 0.581 |  | 0.07 | -0.24;0.37 | 0.679 |  | -0.18 | -0.48;0.11 | 0.218 |  | -0.11 | -0.40;0.18 | 0.460 |
| 3 | 0.05 | -0.26;0.36 | 0.750 |  | 0.14 | -0.16;0.44 | 0.369 |  | 0.07 | -0.17;0.30 | 0.572 |  | 0.06 | -0.25;0.37 | 0.682 |  | -0.19 | -0.48;0.10 | 0.198 |  | -0.12 | -0.41;0.17 | 0.417 |
| 4 | 0.03 | -0.28;0.34 | 0.849 |  | 0.15 | -0.15;0.45 | 0.314 |  | 0.04 | -0.20;0.27 | 0.751 |  | 0.01 | -0.29;0.31 | 0.948 |  | -0.24 | -0.53;0.05 | 0.098 |  | -0.18 | -0.46;0.10 | 0.215 |
| 5 | 0.03 | -0.28;0.34 | 0.850 |  | 0.15 | -0.15;0.45 | 0.310 |  | 0.04 | -0.20;0.27 | 0.754 |  | 0.01 | -0.29;0.31 | 0.951 |  | -0.24 | -0.53;0.05 | 0.098 |  | -0.18 | -0.46;0.10 | 0.216 |
|  |  |  |  |  |  |  |  |  |  |  |  |  |  |  |  |  |  |  |  |  |  |  |  |
| Proliferative retinopathy | | | |  |  |  |  |  |  |  |  |  |  |  |  |  |  |  |  |  |  |  |  |
|  |  | lnMMP-1 |  |  |  | lnMMP-2 |  |  |  | lnMMP-3 |  |  |  | lnMMP-9 |  |  |  | lnMMP-10 |  |  |  | TIMP-1 |  |
| Model | β | 95% CI | p-value |  | β | 95% CI | p-value |  | β | 95% CI | p-value |  | β | 95% CI | p-value |  | β | 95% CI | p-value |  | β | 95% CI | p-value |
| 1 | 0.02 | -0.26;0.30 | 0.897 |  | **0.71** | **0.45;0.96** | **<0.001** |  | **0.49** | **0.28;0.70** | **<0.001** |  | -0.14 | -0.42;0.15 | 0.340 |  | **0.39** | **0.11;0.66** | **0.007** |  | **0.54** | **0.28;0.81** | **<0.001** |
| 2 | -0.15 | -0.48;0.18 | 0.377 |  | **0.36** | **0.06;0.66** | **0.019** |  | 0.17 | -0.06;0.39 | 0.141 |  | -0.14 | -0.48;0.20 | 0.415 |  | 0.02 | -0.29;0.34 | 0.880 |  | 0.09 | -0.20;0.38 | 0.551 |
| 3 | -0.15 | -0.48;0.18 | 0.365 |  | **0.38** | **0.09;0.67** | **0.012** |  | 0.17 | -0.06;0.39 | 0.140 |  | -0.15 | -0.49;0.19 | 0.385 |  | 0.03 | -0.28;0.34 | 0.843 |  | 0.11 | -0.18;0.39 | 0.465 |
| 4 | -0.17 | -0.50;0.17 | 0.328 |  | **0.35** | **0.05;0.64** | **0.024** |  | 0.15 | -0.07;0.37 | 0.189 |  | -0.14 | -0.48;0.20 | 0.415 |  | -0.04 | -0.34;0.27 | 0.816 |  | 0.03 | -0.25;0.31 | 0.826 |
| 5 | -0.18 | -0.51;0.16 | 0.298 |  | **0.38** | **0.08;0.67** | **0.013** |  | 0.15 | -0.08;0.37 | 0.202 |  | -0.22 | -0.55;0.12 | 0.205 |  | -0.04 | -0.34;0.27 | 0.803 |  | 0.05 | -0.23;0.33 | 0.735 |

The standardized regression coefficient β represents the difference in plasma levels of MMPs and TIMP-1 (in SD) in patients with non-proliferative (n=125) or proliferative retinopathy (n=146) vs. those without retinopathy (n=222)

| model 1 | adjusted for age, sex, duration of diabetes and HbA1c | |  | |
| --- | --- | --- | --- | --- |
| model 2 | model 1 + BMI, LDL, HDL, triglycerides, systolic blood pressure, eGFR, smoking, antihypertensive medication, CVD and albuminuria | | | |
| model 3 | model 2 + endothelial dysfunction score |  | | |
| model 4 | model 2 + inflammation score |  | | |
| model 5 | model 2 + endothelial score and inflammation score | | |  |
