# Supplementary material for: Plasma levels of matrix metalloproteinase-2, -3, -10, and tissue inhibitor of metalloproteinase-1 are associated with vascular complications in patients with type 1 diabetes: the EURODIAB Prospective Complications Study
Source: Cardiovasc Diabetol. 2015 Mar 10;14:31. doi: 10.1186/s12933-015-0195-2 (PMC4355971; doi:10.1186/s12933-015-0195-2)
Supplement: Additional file 4: Table S4. — Associations between plasma levels of MMP-1, -2, -3, -9, and -10 and TIMP-1 and markers of low-grade inflammation and endothelial dysfunction. [file 12933_2015_195_MOESM4_ESM.doc]

**Additional table 4. Associations between plasma levels of MMP-1, -2, -3, -9, and -10 and TIMP-1 and markers of low-grade inflammation and endothelial dysfunction**

|  | **Model** | **β** | **95% CI** | **p** |  | **β** | **95% CI** | **p** |  | **β** | **95% CI** | **p** |  | **β** | **95% CI** | **p** |  | **β** | **95% CI** | **p** |
| --- | --- | --- | --- | --- | --- | --- | --- | --- | --- | --- | --- | --- | --- | --- | --- | --- | --- | --- | --- | --- |
| **MMP-1** | 1 | -0.05 | -0.13;0.04 | 0.295 |  | **0.16** | **0.08;0.25** | **<0.001** |  | 0.03 | -0.06;0.12 | 0.507 |  | 0.02 | -0.06;0.11 | 0.579 |  | -0.02 | -0.11;0.06 | 0.592 |
|  | 2 | -0.02 | -0.10;0.07 | 0.673 |  | **0.15** | **0.08;0.23** | **<0.001** |  | 0.04 | -0.05;0.13 | 0.358 |  | 0.03 | -0.06;0.12 | 0.482 |  | -0.03 | -0.12;0.05 | 0.463 |
|  | 3 | -0.02 | -0.10;0.06 | 0.634 |  | **0.14** | **0.07;0.22** | **<0.001** |  | 0.04 | -0.05;0.13 | 0.388 |  | 0.03 | -0.06;0.12 | 0.505 |  | -0.04 | -0.13;0.04 | 0.327 |
| **MMP-2** | 1 | -0.06 | -0.15;0.03 | 0.186 |  | **0.24** | **0.15;0.32** | **<0.001** |  | 0.03 | -0.06;0.12 | 0.542 |  | **-0.11** | **-0.19;-0.02** | **0.018** |  | **0.38** | **0.29;0.46** | **<0.001** |
|  | 2 | -0.07 | -0.16;0.02 | 0.131 |  | **0.13** | **0.05;0.22** | **0.002** |  | 0.00 | -0.10;0.09 | 0.963 |  | **-0.11** | **-0.21;-0.02** | **0.020** |  | **0.31** | **0.22;0.40** | **<0.001** |
|  | 3 | -0.08 | -0.17;0.01 | 0.095 |  | **0.11** | **0.03;0.19** | **0.010** |  | -0.02 | -0.12;0.07 | 0.634 |  | **-0.11** | **-0.20;-0.01** | **0.030** |  | **0.29** | **0.20;0.38** | **<0.001** |
| **MMP-3** | 1 | **0.12** | **0.01;0.23** | **0.031** |  | **0.31** | **0.21;0.41** | **<0.001** |  | **0.14** | **0.03;0.25** | **0.015** |  | -0.09 | -0.19;0.02 | 0.105 |  | **0.20** | **0.09;0.32** | **<0.001** |
|  | 2 | **0.13** | **0.02;0.25** | **0.026** |  | **0.16** | **0.06;0.27** | **0.003** |  | **0.13** | **0.01;0.25** | **0.038** |  | -0.12 | -0.24;0.00 | 0.058 |  | 0.08 | -0.03;0.20 | 0.162 |
|  | 3 | **0.12** | **0.01;0.24** | **0.040** |  | **0.13** | **0.03;0.24** | **0.013** |  | 0.11 | -0.02;0.23 | 0.089 |  | -0.12 | -0.24;0.00 | 0.053 |  | 0.06 | -0.06;0.18 | 0.346 |
| **MMP-9** | 1 | 0.07 | -0.01;0.16 | 0.098 |  | **0.16** | **0.08;0.24** | **<0.001** |  | **0.12** | **0.04;0.21** | **0.006** |  | **0.11** | **0.03;0.19** | **0.010** |  | **-0.14** | **-0.23;-0.06** | **0.001** |
|  | 2 | 0.07 | -0.01;0.15 | 0.080 |  | **0.16** | **0.08;0.23** | **<0.001** |  | **0.13** | **0.04;0.21** | **0.004** |  | **0.11** | **0.02;0.19** | **0.012** |  | **-0.13** | **-0.21;-0.05** | **0.002** |
|  | 3 | 0.07 | -0.01;0.15 | 0.097 |  | **0.15** | **0.08;0.23** | **<0.001** |  | **0.12** | **0.04;0.21** | **0.006** |  | **0.10** | **0.02;0.19** | **0.020** |  | **-0.13** | **-0.22;-0.05** | **0.001** |
| **MMP-10** | 1 | **0.09** | **0.01;0.18** | **0.038** |  | **0.25** | **0.17;0.33** | **<0.001** |  | **0.18** | **0.10;0.27** | **<0.001** |  | 0.06 | -0.02;0.15 | 0.146 |  | **0.12** | **0.03;0.21** | **0.008** |
|  | 2 | **0.13** | **0.04;0.22** | **0.003** |  | **0.18** | **0.10;0.26** | **<0.001** |  | **0.18** | **0.08;0.27** | **<0.001** |  | 0.08 | -0.01;0.17 | 0.095 |  | 0.08 | -0.02;0.17 | 0.101 |
|  | 3 | **0.13** | **0.04;0.22** | **0.004** |  | **0.16** | **0.08;0.24** | **<0.001** |  | **0.17** | **0.08;0.26** | **<0.001** |  | **0.09** | **0.00;0.18** | **0.046** |  | 0.05 | -0.04;0.14 | 0.269 |
| **TIMP-1** | 1 | **0.11** | **0.02;0.20** | **0.014** |  | **0.50** | **0.43;0.57** | **<0.001** |  | **0.14** | **0.05;0.23** | **0.002** |  | **0.10** | **0.02;0.19** | **0.019** |  | **0.25** | **0.17;0.34** | **<0.001** |
|  | 2 | **0.09** | **0.00;0.18** | **0.048** |  | **0.41** | **0.34;0.48** | **<0.001** |  | **0.13** | **0.04;0.23** | **0.007** |  | **0.12** | **0.03;0.21** | **0.013** |  | **0.18** | **0.09;0.27** | **<0.001** |
|  | 3 | 0.09 | 0.00;0.18 | 0.059 |  | **0.40** | **0.32;0.47** | **<0.001** |  | **0.13** | **0.03;0.23** | **0.010** |  | **0.14** | **0.04;0.23** | **0.005** |  | **0.15** | **0.06;0.24** | **0.002** |

CRP TNF-α IL-6 E-Selectin VCAM-1

| β, standardized regression coefficient: indicates increase in the lnCRP, lnTNF-α, lnIL-6, E-selectin and VCAM-1 (in SDs) per 1 SD increase in lnMMP-1, -2, -3, -9, -10 or TIMP-1. MMP: matrix metalloproteinase; CRP, C-reactive protein; TNF-α, tumor necrosis factor-α; IL-6, interleukin-6; VCAM-1, vascular cellular adhesion molecule-1 |
| --- |
| Model 1: adjusted for age, sex, duration of diabetes and HbA1c |
| Model 2: model 1 + BMI, triglycerides, LDL, HDL, systolic blood pressure, eGFR, smoking and  antihypertensive medication |
| Model 3: model 2 + CVD, albuminuria and retinopathy |
